# Supplementary material for: Using machine learning to predict persecutory beliefs based on aetiological models of delusions identified in a systematic literature search
Source: Commun Psychol. 2025 Sep 29;3:138. doi: 10.1038/s44271-025-00311-9 (PMC12479767; doi:10.1038/s44271-025-00311-9)
Supplement: Supplementary file 2 — Supplementary Information [file 44271_2025_311_MOESM2_ESM.pdf]

Using machine learning to predict persecutory beliefs based on aetiological models of delusions identified in a systematic literature search

- Supplementary Material -

Saskia Denecke[a], Felix Strakeljahn[a], Antonia Bott[a], & Tania M. Lincoln[a]

[a] Clinical Psychology and Psychotherapy, University of Hamburg

Author Note

Saskia Denecke https://orcid.org/0000-0001-7840-7909

Antonia Bott https://orcid.org/0000-0003-4670-2844

Felix Strakeljahn https://orcid.org/0000-0001-7803-4401

Tania M. Lincoln https://orcid.org/0000-0002-6674-2440

Correspondence should be addressed to Saskia Denecke, University of Hamburg, Von-Melle-Park 5, 20146 Hamburg, Germany. Email: Saskia.denecke@outlook.com

Supplement 1

Supplementary information on the extraction of predictors.

Table S1

Description of Excluded Predictors and their Reason for Exclusion

Predictor	Description	Reason for exclusion
Aberrant Semantic Processing	Aberrant processing of (idiosyncratic) semantic memory or a hyperactive associative network (i.e., overly active semantic associations between concepts).	No validated measure available
Attentional Threat Avoidance	A controlled attentional bias away from threat following automatic increased attention to threat information.	Primarily a maintenance factor
Conditional Inattention	A Lack of conditioned inattention towards redundant information.	No validated and feasible measure available
Dopamine Dysregulation	Dysregulated dopaminergic system	Not feasible in an online setting.
Dysregulated Correlation Detection	Aberrant detection of correlations (e.g., perceiving random co-occurrences to be correlated).	No validated measure available
Hypersalience of Evidence-Hypothesis Matches	Overweighing evidence supporting once hypothesis compared to evidence contradicting it.	No validated measure available
Impaired Conflict Processing	A neurophysiological deficit in detecting conflicts (e.g., conflicting information).	No validated measure available
Neurological Abnormalities	Structural and functional abnormalities in different brain regions.	Not feasible in an online setting.
Partial Reinforcement	A conditioning process wherein behaviour is intermittently reinforced.	Primarily a maintenance factor
Safety Behaviours	Behaviours that are thought to protect oneself from unpleasant experiences or emotions.	Primarily a maintenance factor
Schizotypy/ Paranoid Personality	Personality characteristics related to psychosis	Conceptual overlap with persecutory beliefs /delusions

Supplement 2

Sample characteristics, descriptives of predictors and correlations between the predicted outcomes.

Table S2

Self-reported Mental Health Diagnoses per Quota

Variable	Average N = 84[1]	Elevated N = 84[1]	Moderately severe N = 84[1]	(Very) severe N = 84[1]
Diagnosis of Psychosis	0 (0.0%)	0 (0.0%)	0 (0.0%)	2 (2.4%)
Diagnosis of Depression	13 (15.5%)	16 (19.0%)	16 (19.0%)	26 (31.0%)
Diagnosis of Anxiety Disorder	9 (10.7%)	17 (20.2%)	18 (21.4%)	31 (36.9%)
Diagnosis of OCD	2 (2.4%)	2 (2.4%)	1 (1.2%)	2 (2.4%)
Diagnosis of PTSD	1 (1.2%)	0 (0.0%)	1 (1.2%)	2 (2.4%)
Diagnosis of Autism	0 (0.0%)	0 (0.0%)	1 (1.2%)	1 (1.2%)
Diagnosis of BPD	2 (2.4%)	1 (1.2%)	1 (1.2%)	0 (0.0%)
Diagnosis of Eating Disorder	0 (0.0%)	1 (1.2%)	2 (2.4%)	1 (1.2%)
Diagnosis of Another Disorder	0 (0.0%)	2 (2.4%)	1 (1.2%)	0 (0.0%)

[1]n = 84, N = 336

Note. OCD = obsessive-compulsive disorder, PTSD = post-traumatic stress disorder, BPD = Borderline personality disorder, R-GPTS = Revised Green Paranoid Thoughts Scale[10]

Table S3

Means, Standard Deviations, and Theoretical Scale Range of the Predictor Variables

Predictor	Mean	SD	Theoretical Scale Range
**Aberrant Salience**<br>Aberrant Salience Inventory (ASI)[11]	5.43	2.96	0 - 10
**Anxiety**<br>Depression, Anxiety, and Stress Scale (DASS-21)[12]	9.27	7.85	0 - 42
**Attentional Bias**<br>Emotional Stroop Task[13]	-10.98	49.77	-130 - 151[a]
**Bias Against Disconfirmatory Evidence (BADE)**<br>Bias Against Disconfirmatory Evidence (BADE) Task[14]	-4.72	1.92	-10 – 0[a]
**Cognitive Fusion**<br>Cognitive Fusion Questionnaire-7 (CFQ-7)[15]	28.37	10.51	7 - 49
**Depression**<br>Depression, Anxiety, and Stress Scale (DASS-21)[12]	13.83	9.94	0 - 42
**Dichotomous Thinking**<br>Cognitive Biases in Psychosis Questionnaire (CBQp)[16]	7.23	1.71	5 - 15
**Discrimination**[17]	1.06	1.19	0 - 7
**Emotion Recognition**<br>(Reading the mind in the eyes test - short form)[18]	7.89	1.46	0 - 10
**Emotion Regulation**<br>Difficulties in Emotion Regulation Scale (DERS-16)[19]	40.87	14.80	16 - 80
**Externalising Bias**<br>Internal, Personal and Situational Attributions Questionnaire (IPSAQ)[20]	12.26	110.04	-395 – 514[a]
**Genetics**<br>Family History Screen (based on ref. [21])	0.12	0.32	0/1
**Guilt**<br>Self-Report Instrument for the Assessment of Emotion-Specific Regulation Skills (ERSQ-ES)[22]	1.28	1.11	0 - 4
**Hallucinations**<br>Launay-Slade Hallucination Scale- Modified II (LSHS-II)[23]	22.67	15.02	0 - 64
**Intelligence**<br>Hagen Matrices Test - 6 item version (HMT-S)[24]	3.22	1.56	0 - 6
**Intolerance of Ambiguity**<br>Intolerance of Ambiguity Scale[25]	22.10	10.81	12 - 60
**Jumping to Conclusions Bias (JTC)**<br>Beads Task[26]	4.25	2.49	1 - 8
**Liberal Acceptance**<br>BADE Task[14]	7.48	1.34	3.88 – 10[a]
**Life Purpose – Search**<br>Meaning in Life Questionnaire (MLQ)[27]	22.76	7.04	5 - 35
**Life Purpose – Presence**<br>Meaning in Life Questionnaire (MLQ)[27]	19.55	7.28	5 - 35

Predictor	Mean	SD	Theoretical Scale Range
**Likelihood Weight**<br>Beads Estimation Task (adapted from ref. [28])	1.02	1.64	0 - 20
**Minority Status**[17]	0.86	0.91	0 - 5
**Negative Beliefs about Mistrust**<br>Beliefs about Paranoia Scale (BaPS - negative subscale)[29]	11.36	4.26	6 - 24
**Negative Other Schemas**<br>Brief Core Schema Scale (BCSS)[30]	7.62	5.22	0 - 24
**Neurodevelopmental Hazards**<br>Murray-Lewis Obstetric Complications Scale (MLOCS)[31]	0.37	0.48	0/1[b]
**Ostracism**<br>Ostracism Short Scale (OSS)[32]	12.08	6.13	4 - 28
**Overconfidence in Judgements**<br>Beck Cognitive Insight Scale (BCIS - self-certainty subscale)[33]	7.79	2.93	0 - 18
**Perceived Control**<br>Perceived Control Scale (PCS)[34]	20.00	7.29	5 - 35
**Personalising Bias**<br>Internal, Personal and Situational Attributions Questionnaire (IPSAQ)[20]	0.56	0.14	0 - 1
**Physical Health**<br>Somatic Symptom Scale 8 (SSS-8)[35]	10.75	6.16	0 - 32
**Positive Beliefs about Mistrust**<br>Beliefs about Paranoia Scale (BaPS - survival subscale)[29]	15.29	4.15	6 - 24
**Prior Weight**<br>Beads Estimation Task (adapted from ref. [28])	0.94	0.26	0 – 1.42[a]
**Reasoning – Analytical**<br>Cognitive Reflection Task (CRT)[36,37]	3.89	1.99	0 - 8
**Reasoning – Intuitive**<br>Cognitive Reflection Task (CRT)[36,37]	2.58	1.65	0 - 8
**Self-Awareness**<br>Private Self-Consciousness Scale – Revised (P-SCS)[38]	15.32	5.36	0 - 27
**Self-Beliefs – Positive**<br>Brief Core Schema Scale (BCSS)[30]	10.12	5.09	0 - 24
**Self-Beliefs – Negative**<br>Brief Core Schema Scale (BCSS)[30]	5.27	4.79	0 - 24
**Sensory Deficit**<br>(based on ref. [39])	0.60	0.78	0 - 6
**Shame**<br>Self-Report Instrument for the Assessment of Emotion-Specific Regulation Skills (ERSQ-ES)[22]	1.09	1.09	0 - 4
**Sleep Problems**<br>Insomnia Severity Index (ISI)[40]	10.61	5.94	0 - 28

Predictor	Mean	SD	Theoretical Scale Range
**Social Anxiety**<br>Brief Fear of Negative Evaluation (BFNE-II)[41]	17.14	9.12	0 - 32
**Social Cognition**<br>Hinting Task[42]	15.33	2.73	0 - 10
**Social Support**<br>Interpersonal Support Evaluation List short form (ISEL)[43]	33.39	8.04	12 - 48
**Socioeconomic Status**<br>(based on ref. [44])	7.18	2.24	2 - 13
**Stress**<br>Depression, Anxiety, and Stress Scale (DASS-21)[12]	15.21	9.20	0 - 42
**Stress Reactivity**<br>Arousal Predisposition Scale (APS)[45]	35.58	8.29	12 - 60
**Substance Use**<br>Substance Use Questionnaire (SUQ)[46]	14.21	14.60	0 - 240[c]
**Threat Anticipation**<br>Negative Events Scale (NES)[47,48]	32.69	12.41	7 - 70
**Trauma**<br>Childhood Abuse Questionnaire[49]	4.33	4.61	0 - 20
**Trust**<br>Interpersonal Trust Short Scale (KUSIV3)[50]	3.17	0.80	5 - 15
**Urbanicity during Childhood**<br>(based on previous studies[51])	2.98	1.48	1 - 7
**Urbanicity at Present**<br>(based on previous studies[51])	3.15	1.52	1 - 7
**Working Memory**<br>Digit Span Backwards task[52]	9.91	3.20	0 - 16
**Worry Thinking Style**<br>3-item Penn State Worry Questionnaire (PSWQ-3)[53]	9.12	3.50	3 - 15

[a] Observed minimum – maximum range

[b] Dichotomised as 1 (yes, any) or 0 (none)

[c] The total score is the sum of the frequency multiplied by the quantity of each substance category.

Figure S1

Absolute Correlations between Predicted Outcomes

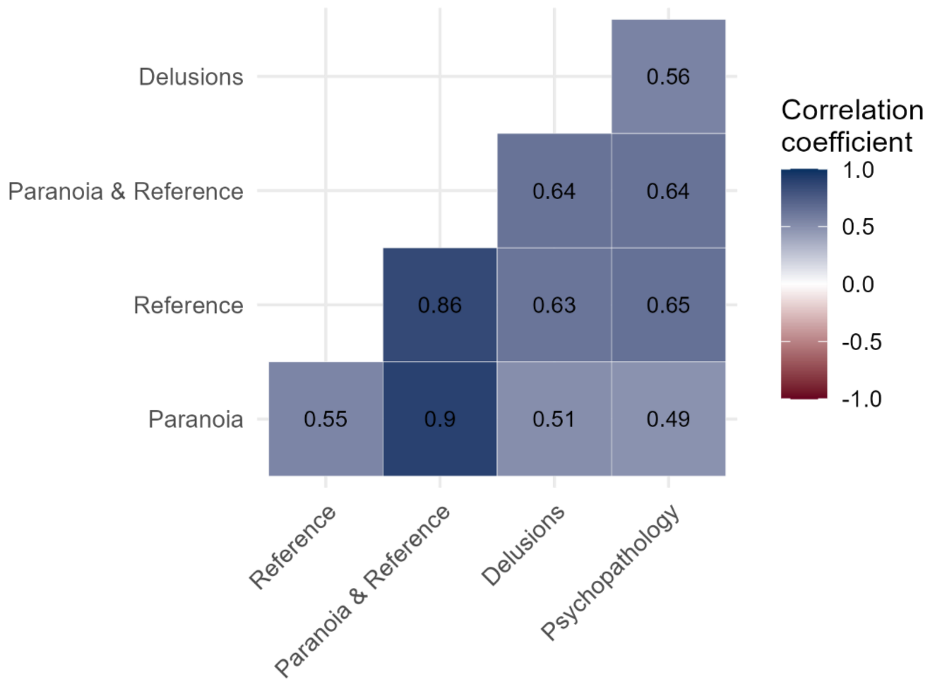

Supplement 3

Hyperparameter space specifications, scatterplots of the observed and predicted values, and beeswarm plots of the SHAP values for the models predicting the full Revised Green Paranoid Thoughts Scale (R-GPTS Persecution and Reference), the Peters Delusion Inventory, the Symptom Checklist (SCL-K-9), and the exploratory models.

Table S4

Hyperparameter Space used for Hyperparameter Tuning

Hyperparameter	Possible values
Number of trees	50, 100, 200
Max. depth	2 - 30 in steps of two
Max. features per split	20%, 40%, 60%, 80%
Min. samples per leaf	2 - 50 in steps of two

Figure S2

Scatterplots of the Observed and Predicted Scores for A) Persecutory Beliefs, B) Persecutory Beliefs and Ideas of Reference, C) Delusions in General, and D) General Psychopathology

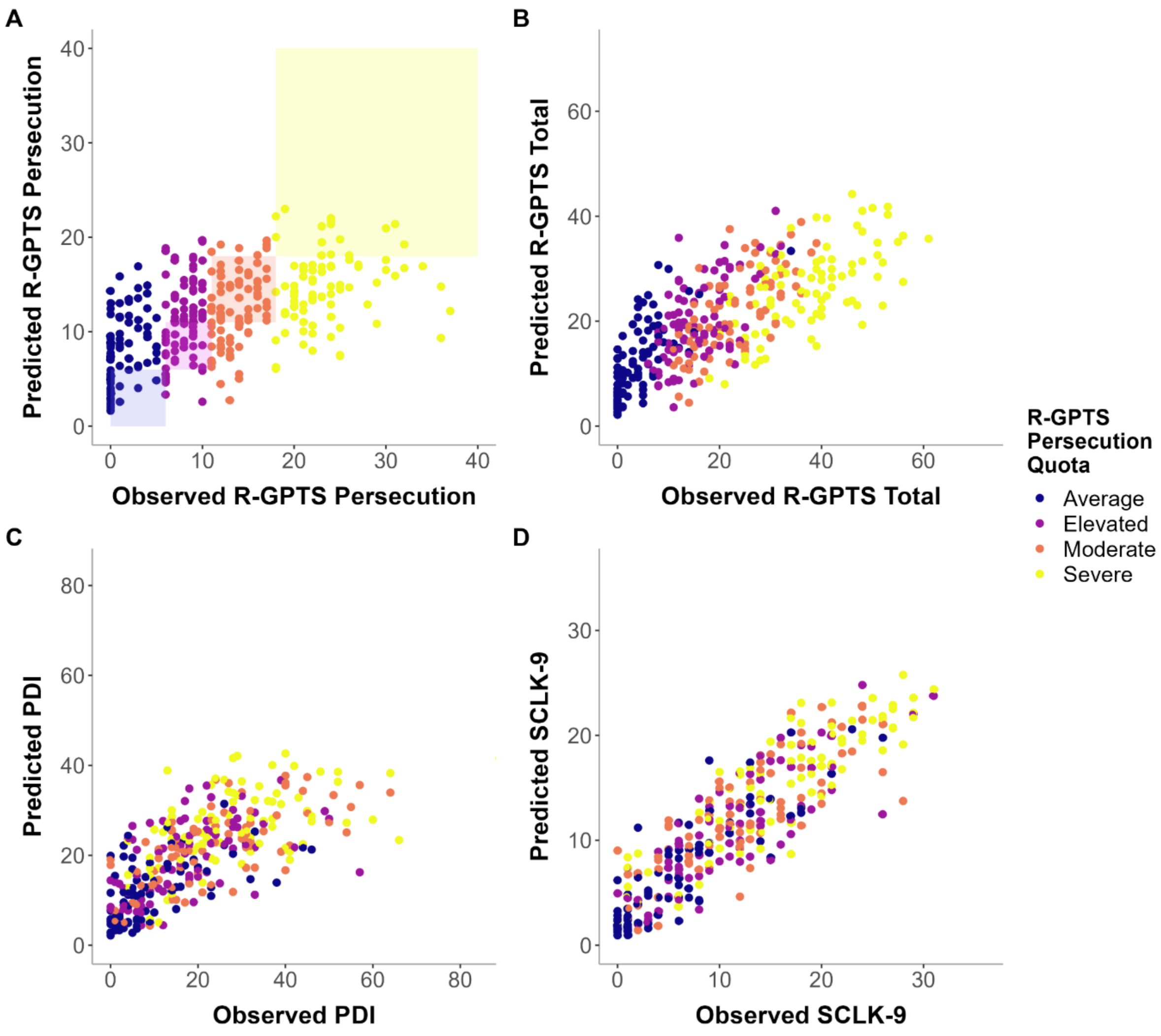

Note. R-GPTS = Revised Green Paranoid Thoughts Scale, PDI = Peters Delusion Inventory, SCL-K-9 = Symptom Checklist short form. The colour of the dots indicates the observed and assigned quota. A: The coloured rectangles indicate the area of the quota range.

Figure S3

Scatterplots of the Observed and Predicted Scores for Persecutory Beliefs A) Including the Top Ten Predictors only and B) Excluding the Top Ten Predictors

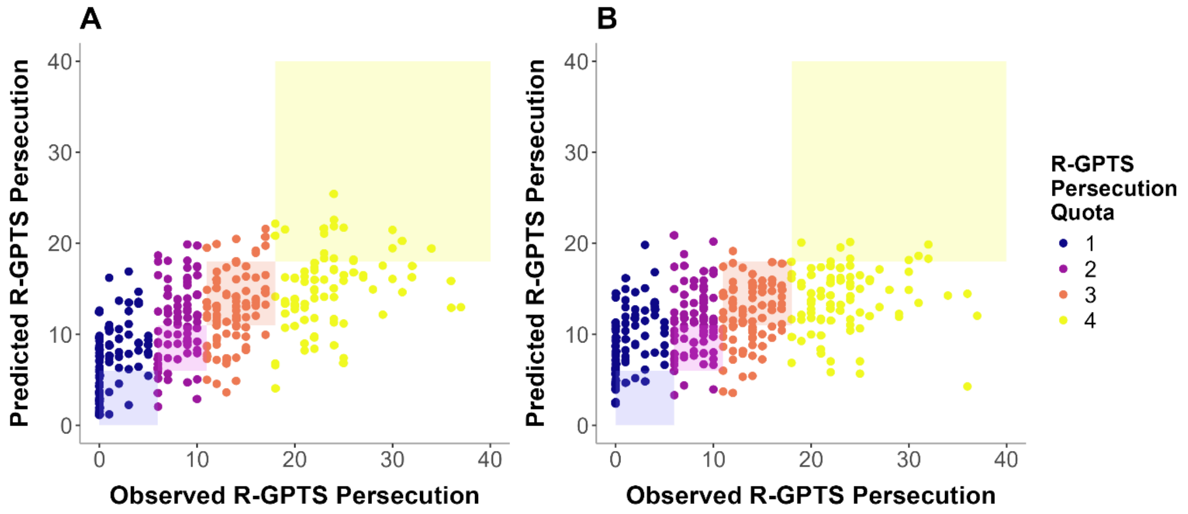

Note. R-GPTS = Revised Green Paranoid Thoughts Scale. The colour of the dots indicates the observed and assigned quota. The coloured rectangles indicate the area of the quota range.

Figure S4

Beeswarm Plots of the SHAP-values for the Reduced Models Predicting Persecutory Beliefs A) Including the Top Ten Predictors only and B) Excluding the Top Ten Predictors

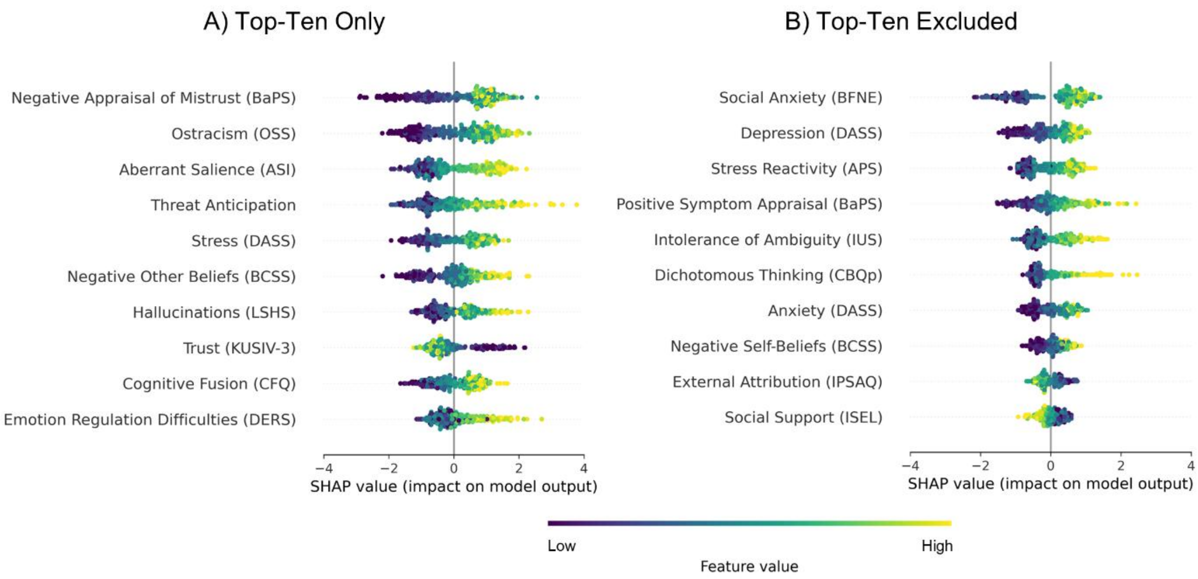

Note. The ten predictors with the highest SHAP values are presented in descending order. Each dot represents a participant, with its position on the x-axis indicating how much that variable influences the model's prediction of the individual score. When several dots overlap at the same position, they stack up to illustrate density. The colour of each point indicates the value of the predictor variable (i.e., feature value). Positive SHAP values indicate a change in the expected model prediction towards higher levels of the outcome variable; negative SHAP values indicate a change in the expected model prediction towards lower levels of the outcome variable.

Table S5

Nested Cross-Validation results of Random Forests on Categorical Persecutory Beliefs Outcomes (R-GPTS Persecution)

**Model**	**Precision**	**Recall**	**Accuracy**
Binary Classification Model			68%
High R-GPTS Persecution (≥ 11)	67%	69%	
Low R-GPTS Persecution (< 11)	68%	67%	
Multiclass Classification Model			41%
Average Quota (0–5)	52%	64%	
Elevated Quota (6–10)	27%	21%	
Moderately Severe Quota (11–17)	36%	30%	
Severe and Very Severe Quota (≥ 18)	44%	50%	

Note. R-GPTS = Revised Green Paranoid Thoughts Scale, Precision = Number of people correctly predicted by the model to belong to this quota (true positives) divided by the total number of people predicted by the model as belonging to this quota (true positives + false positives; for dichotomized outcomes, this is often referred to as positive predictive value), Recall = Number of people correctly predicted by the model to belong to this quota (true positives) divided by the total number of people who actually belong to this quota in the dataset (true positives + false negatives; for dichotomized outcomes, this is also referred to as sensitivity.

Figure S5

Beeswarm Plot Displaying the Ten Predictors with the Highest SHAP-Values for the Binary Prediction Model of the R-GPTS Clinical Cut-Off (≥ 11)

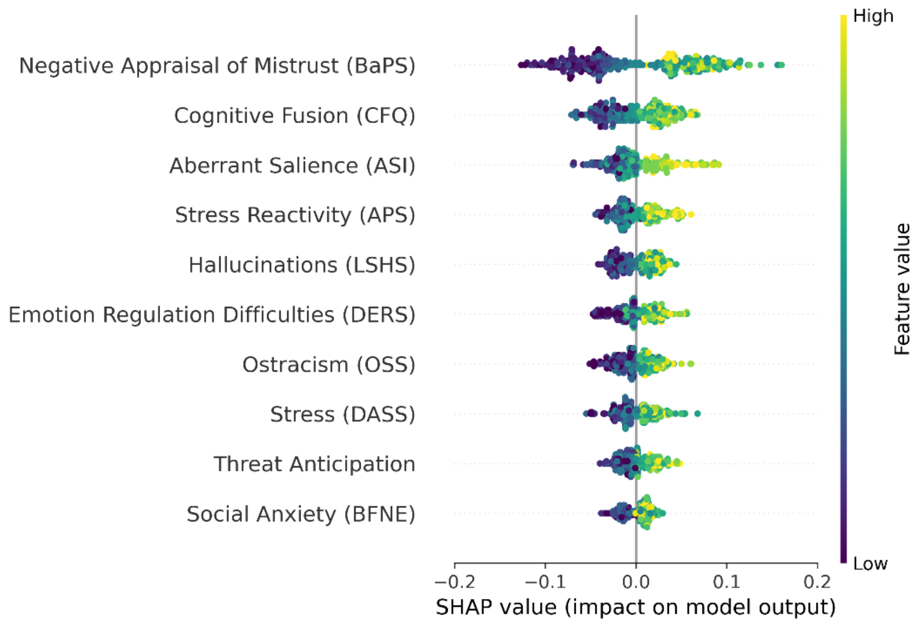

Note. A positive SHAP value indicates that the variable influences the model to predict someone scoring above the clinical cut-off of the R-GPTS Persecution (≥ 11). The ten predictors with the highest SHAP values are presented in descending order. Each dot represents a participant, with its position on the x-axis indicating how much that variable influences the model's prediction of the individual score. When several dots overlap at the same position, they stack up to illustrate density. The colour of each point indicates the value of the predictor variable (i.e., feature value).

Figure S6

Beeswarm Plots Displaying the Ten Predictors with the Highest SHAP-Values for the Multiclass Classification Model of the Four R-GPTS Quotas

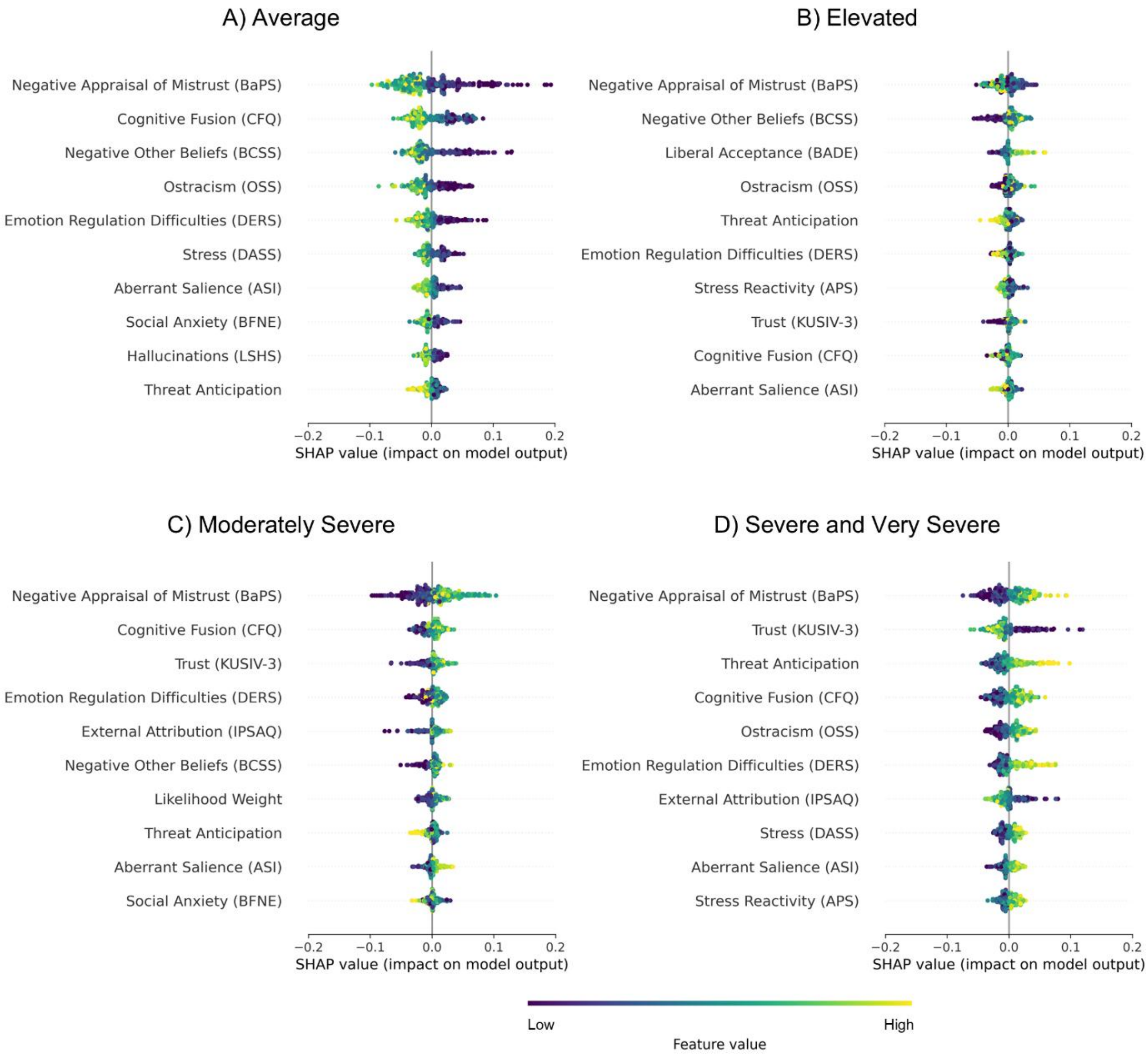

Note. The ten predictors with the highest SHAP values are presented in descending order. Each dot represents a participant, with its position on the x-axis indicating how much that variable influences the model's prediction of the individual score. When several dots overlap at the same position, they stack up to illustrate density. The colour of each point indicates the value of the predictor variable (i.e., feature value).

Supplementary References

1. Beck, A. T., Rector, N. A., Stolar, N. & Grant, P. *Schizophrenia: Cognitive Theory, Research, and Therapy*. (The Guilford Press, New York, 2009).
2. Denève, S. & Jardri, R. Circular inference: mistaken belief, misplaced trust. *Curr. Opin. Behav. Sci.* **11**, 40–48 (2016).
3. Feeney, E. J., Groman, S. M., Taylor, J. R. & Corlett, P. R. Explaining delusions: Reducing uncertainty through basic and computational neuroscience. *Schizophr. Bull.* **43**, 263–272 (2017).
4. Friston, K., Brown, H. R., Siemerkus, J. & Stephan, K. E. The dysconnection hypothesis. *Schizophr. Res.* **176**, 83–94 (2016).
5. Greenaway, K. H., Haslam, S. A. & Bingley, W. Are "they" out to get me? A social identity model of paranoia. *Group Process. Intergroup Relat.* **22**, 984–1001 (2019).
6. Morrison, A. P. The interpretation of intrusions in psychosis: An integrative cognitive approach to hallucinations and delusions. *Behav. Cogn. Psychother.* **29**, 257–276 (2001).
7. Newman-Taylor, K. *et al.* Cognitive mechanisms in cannabis-related paranoia; Initial testing and model proposal. *Psychosis* **12**, 314–327 (2020).
8. Preti, A. & Cella, M. Paranoid thinking as a heuristic: Paranoid thinking as a heuristic. *Early Interv. Psychiatry* **4**, 263–266 (2010).
9. Denecke, S., Schönig, S. N., Bott, A., Faße, J. L. & Lincoln, T. M. Bridging perspectives - A review and synthesis of 53 theoretical models of delusions. *Clin. Psychol. Rev.* **114**, 102510 (2024).
10. Freeman, D. *et al.* The revised Green *et al.* , Paranoid Thoughts Scale (R-GPTS): psychometric properties, severity ranges, and clinical cut-offs. *Psychol. Med.* **51**, 244–253 (2021).
11. Cicero, D. C., Kerns, J. G. & McCarthy, D. M. The Aberrant Salience Inventory: A new measure of psychosis proneness. *Psychol. Assess.* **22**, 688–701 (2010).

12. Lovibond, P. F. & Lovibond, S. H. The structure of negative emotional states: Comparison of the Depression Anxiety Stress Scales (DASS) with the Beck Depression and Anxiety Inventories. *Behav. Res. Ther.* **33**, 335–343 (1995).
13. Aleksandrowicz, A. *et al.* Frontal brain activity in individuals at risk for schizophrenic psychosis and bipolar disorder during the emotional Stroop task – an fNIRS study. *NeuroImage Clin.* **26**, 102232 (2020).
14. Veckenstedt, R. *et al.* Incorrigibility, jumping to conclusions, and decision threshold in schizophrenia. *Cognit. Neuropsychiatry* **16**, 174–192 (2011).
15. Gillanders, D. T. *et al.* The development and initial validation of the Cognitive Fusion Questionnaire. *Behav. Ther.* **45**, 83–101 (2014).
16. Peters, E. R. *et al.* Cognitive biases questionnaire for psychosis. *Schizophr. Bull.* **40**, 300–313 (2014).
17. Janssen, I. *et al.* Discrimination and delusional ideation. *Br. J. Psychiatry* **182**, 71–76 (2003).
18. Olderbak, S. *et al.* A psychometric analysis of the reading the mind in the eyes test: toward a brief form for research and applied settings. *Front. Psychol.* **6**, (2015).
19. Bjureberg, J. *et al.* Development and validation of a brief version of the Difficulties in Emotion Regulation Scale: The DERS-16. *J. Psychopathol. Behav. Assess.* **38**, 284–296 (2016).
20. Kinderman, P. & Bentall, R. P. A new measure of causal locus: the internal, personal and situational attributions questionnaire. *Personal. Individ. Differ.* **20**, 261–264 (1996).
21. Weissman, M. M. Brief screening for family psychiatric history: The Family History Screen. *Arch. Gen. Psychiatry* **57**, 675–682 (2000).
22. Ebert, D. D., Christ, O. & Berking, M. Entwicklung und Validierung eines Fragebogens zur emotionsspezifischen Selbsteinschätzung emotionaler Kompetenzen (SEK-ES). *Diagnostica* **59**, 17–32 (2013).
23. Larøi, F. & Van Der Linden, M. Nonclinical participants' reports of hallucinatory experiences. *Can. J. Behav. Sci. Rev. Can. Sci. Comport.* **37**, 33–43 (2005).

24. Heydasch, T., Haubrich, J. & Renner, K.-H. The short version of the Hagen Matrices Test (HMT-S): 6-item induction intelligence test. *methods* **data**, 26 Pages (2017).

25. Carleton, R. N., Norton, M. A. P. J. & Asmundson, G. J. G. Fearing the unknown: A short version of the Intolerance of Uncertainty Scale. *J. Anxiety Disord.* **21**, 105–117 (2007).

26. Huq, S. F., Garety, P. A. & Hemsley, D. R. Probabilistic judgements in deluded and non-deluded subjects. *Q. J. Exp. Psychol. Sect. A* **40**, 801–812 (1988).

27. Steger, M. F., Frazier, P., Oishi, S. & Kaler, M. The meaning in life questionnaire: Assessing the presence of and search for meaning in life. *J. Couns. Psychol.* **53**, 80–93 (2006).

28. Baker, S. C., Konova, A. B., Daw, N. D. & Horga, G. A distinct inferential mechanism for delusions in schizophrenia. *Brain* **142**, 1797–1812 (2019).

29. Gumley, A. I., Gillan, K., Morrison, A. P. & Schwannauer, M. The development and validation of the Beliefs about Paranoia Scale (short form). *Behav. Cogn. Psychother.* **39**, 35–53 (2011).

30. Fowler, D. *et al.* The Brief Core Schema Scales (BCSS): psychometric properties and associations with paranoia and grandiosity in non-clinical and psychosis samples. *Psychol. Med.* **36**, 749–759 (2006).

31. Lewis, S. W. & Murray, R. M. Obstetric complications, neurodevelopmental deviance, and risk of schizophrenia. *J. Psychiatr. Res.* **21**, 413–421 (1987).

32. Rudert, S. C., Keller, M. D., Hales, A. H., Walker, M. & Greifeneder, R. Who gets ostracized? A personality perspective on risk and protective factors of ostracism. *J. Pers. Soc. Psychol.* **118**, 1247–1268 (2020).

33. Beck, A. T. A new instrument for measuring insight: the Beck Cognitive Insight Scale. *Schizophr. Res.* **68**, 319–329 (2004).

34. Whitaker, D. J., Miller, K. S. & Clark, L. F. Reconceptualizing adolescent sexual behavior: Beyond did they or didn't they? *Fam. Plann. Perspect.* **32**, 111 (2000).

35. Gierk, B. *et al.* The Somatic Symptom Scale–8 (SSS-8): A brief measure of somatic symptom burden. *JAMA Intern. Med.* **174**, 399 (2014).

36. Thomson, K. S. & Oppenheimer, D. M. Investigating an alternate form of the cognitive reflection test. *Judgm. Decis. Mak.* **11**, 15 (2016).

37. Toplak, M. E., West, R. F. & Stanovich, K. E. Assessing miserly information processing: An expansion of the Cognitive Reflection Test. *Think. Reason.* **20**, 147–168 (2014).

38. Scheier, M. F. & Carver, C. S. The Self-Consciousness Scale: A revised version for use with general populations. *J. Appl. Soc. Psychol.* **15**, 687–699 (1985).

39. Capella-McDonnall, M. E. The effects of single and dual sensory loss on symptoms of depression in the elderly. *Int. J. Geriatr. Psychiatry* **20**, 855–861 (2005).

40. Morin, C. M. *Insomnia: Psychological Assessment and Management*. (Guilford Press, New York, 1993).

41. Carleton, R. N., Collimore, K. C. & Asmundson, G. J. G. Social anxiety and fear of negative evaluation: Construct validity of the BFNE-II. *J. Anxiety Disord.* **21**, 131–141 (2007).

42. Corcoran, R., Mercer, G. & Frith, C. D. Schizophrenia, symptomatology and social inference: Investigating “theory of mind” in people with schizophrenia. *Schizophr. Res.* **17**, 5–13 (1995).

43. Payne, T. J. *et al.* Psychometric evaluation of the Interpersonal Support Evaluation List–short form in the ARIC study cohort. *SAGE Open* **2**, 215824401246192 (2012).

44. Lampert, T. & Kroll, L. E. Die Messung des sozioökonomischen Status in sozialepidemiologischen Studien. in *Gesundheitliche Ungleichheit: Grundlagen, Probleme, Perspektiven* (VS, Verl. für Sozialwissenschaften, Wiesbaden, 2009).

45. Coren, S. Prediction of insomnia from arousability predisposition scores: Scale development and cross-validation. *Behav. Res. Ther.* **26**, 415–420 (1988).

46. Hildebrandt, M. K., Dieterich, R. & Endrass, T. Disentangling substance use and related problems: urgency predicts substance-related problems beyond the degree of use. *BMC Psychiatry* **21**, 242 (2021).

47. Kaney, S., Bowen-Jones, K., Dewey, M. E. & Bentall, R. P. Two predictions about paranoid ideation: Deluded, depressed and normal participants’ subjective frequency and

consensus judgments for positive, neutral and negative events. *Br. J. Clin. Psychol.* **36**, 349–364 (1997).

48. Reininghaus, U. *et al.* Liberal Acceptance Bias, Momentary Aberrant Salience, and Psychosis: An Experimental Experience Sampling Study. *Schizophr. Bull.* **45**, 871–882 (2019).
49. Janssen, I. *et al.* Childhood abuse as a risk factor for psychotic experiences. *Acta Psychiatr. Scand.* **109**, 38–45 (2004).
50. Nießen, D., Beierlein, C., Rammstedt, B. & Lechner, C. M. An English-language adaptation of the Interpersonal Trust Short Scale (KUSIV3). *Meas. Instrum. Soc. Sci.* **2**, 10 (2020).
51. Lincoln, T. M. *et al.* Taking a machine learning approach to optimize prediction of vaccine hesitancy in high income countries. *Sci. Rep.* **12**, 2055 (2022).
52. *Wechsler Adult Intelligence Scale (WAIS-IV). Manual 1: Grundlagen, Testauswertung und Interpretation / Übersetzung und Adaptation der WAIS-IV von David Wechsler ; F. Petermann (Hrsg.)*. (Pearson, Frankfurt am Main, 2014).
53. Berle, D. *et al.* Preliminary validation of an ultra-brief version of the Penn State Worry Questionnaire. *Clin. Psychol. Psychother.* **18**, 339–346 (2011).
