# Supplementary material for: Using machine learning to predict persecutory beliefs based on aetiological models of delusions identified in a systematic literature search
Source: Commun Psychol. 2025 Sep 29;3:138. doi: 10.1038/s44271-025-00311-9 (PMC12479767; doi:10.1038/s44271-025-00311-9)
Supplement: Supplementary file 3 — Reporting Summary [file 44271_2025_311_MOESM3_ESM.pdf]

nature portfolio

Corresponding author(s): Denecke, Saskia

Last updated by author(s): 30.07.2025



# Reporting Summary

Nature Portfolio wishes to improve the reproducibility of the work that we publish. This form provides structure for consistency and transparency in reporting. For further information on Nature Portfolio policies, see our Editorial Policies and the Editorial Policy Checklist.

## Statistics

For all statistical analyses, confirm that the following items are present in the figure legend, table legend, main text, or Methods section.

| n/a | Confirmed | |
|---|---|---|
| ☐ | ☒ | The exact sample size (*n*) for each experimental group/condition, given as a discrete number and unit of measurement |
| ☒ | ☐ | A statement on whether measurements were taken from distinct samples or whether the same sample was measured repeatedly |
| ☐ | ☒ | The statistical test(s) used AND whether they are one- or two-sided<br>*Only common tests should be described solely by name; describe more complex techniques in the Methods section.* |
| ☐ | ☒ | A description of all covariates tested |
| ☐ | ☒ | A description of any assumptions or corrections, such as tests of normality and adjustment for multiple comparisons |
| ☐ | ☒ | A full description of the statistical parameters including central tendency (e.g. means) or other basic estimates (e.g. regression coefficient) AND variation (e.g. standard deviation) or associated estimates of uncertainty (e.g. confidence intervals) |
| ☒ | ☐ | For null hypothesis testing, the test statistic (e.g. *F*, *t*, *r*) with confidence intervals, effect sizes, degrees of freedom and *P* value noted<br>*Give P values as exact values whenever suitable.* |
| ☒ | ☐ | For Bayesian analysis, information on the choice of priors and Markov chain Monte Carlo settings |
| ☒ | ☐ | For hierarchical and complex designs, identification of the appropriate level for tests and full reporting of outcomes |
| ☒ | ☐ | Estimates of effect sizes (e.g. Cohen's *d*, Pearson's *r*), indicating how they were calculated |

*Our web collection on statistics for biologists contains articles on many of the points above.*

## Software and code

Policy information about availability of computer code

| | |
|---|---|
| Data collection | The data was collected using the open-source platform PsyToolkit. The code was written by the authours. For one of the tasks (Beads estimation task), we adapted code that was shared with us by Brandon Ashinoff, which we acknowledge in the acknowledgements. |
| Data analysis | The data was preprocessed using R (version 4.4.3) and MATLAB (version R2022b) and analysed using Python (version 3.8.6) code written by the authors. The code will be made available on the OSF under a CC BY-NC-SA 4.0 license upon publication of the article. |

For manuscripts utilizing custom algorithms or software that are central to the research but not yet described in published literature, software must be made available to editors and reviewers. We strongly encourage code deposition in a community repository (e.g. GitHub). See the Nature Portfolio guidelines for submitting code & software for further information.

## Data

Policy information about availability of data

All manuscripts must include a data availability statement. This statement should provide the following information, where applicable:

- Accession codes, unique identifiers, or web links for publicly available datasets
- A description of any restrictions on data availability
- For clinical datasets or third party data, please ensure that the statement adheres to our policy

The de-identified data for the reported analyses is available on the OSF (https://osf.io/7jexv/; doi: 10.17605/OSF.IO/7JEXV).

## Research involving human participants, their data, or biological material

Policy information about studies with human participants or human data. See also policy information about sex, gender (identity/presentation), and sexual orientation and race, ethnicity and racism.

| | |
|---|---|
| Reporting on sex and gender | Both sex assigned at birth and self-identified gender were assessed in this study. Only sex assigned at birth was used as a predictor in the machine learning analysis.<br><br>Regarding self-identified gender, in our sample, n = 170 self-identified as male, n =161 as female, n = 4 as non-binary and n = 1 indicated "none of these apply". |
| Reporting on race, ethnicity, or other socially relevant groupings | Data on the participants' self-reported ethnicity was provided by Prolific. On Prolific, participants self-reported their ethnicity using a mutliple choice item. |
| Population characteristics | Participant self-reported their age and past or current mental health diagnoses. The mean age in the sample was 39.5 (SD = 12.9). In the sample, 0.6% reported aving received a disgnosis of a psychotic disorder (e.g., schizophrenia, schizooaffective disorder) and 32.7% reported having received any mental health diagnosis (e.g., depression, anxiety disorder, bipolar disorder). |
| Recruitment | Participants were recruited via the online recruitment platform Prolific. Individuals who participated in our study were self-selected, literate, had access to the Internet, and were able to complete the relatively long survey, which may not be representative of individuals in acute phases of psychosis. |
| Ethics oversight | Local ethics committee of the University of Hamburg |

Note that full information on the approval of the study protocol must also be provided in the manuscript.

# Field-specific reporting

Please select the one below that is the best fit for your research. If you are not sure, read the appropriate sections before making your selection.

☐ Life sciences ☒ Behavioural & social sciences ☐ Ecological, evolutionary & environmental sciences

For a reference copy of the document with all sections, see nature.com/documents/nr-reporting-summary-flat.pdf

# Behavioural & social sciences study design

All studies must disclose on these points even when the disclosure is negative.

| | |
|---|---|
| Study description | The study is a cross-sectional quantitative online survey. |
| Research sample | Participants were recruited via the online recruitment platform Prolific (www.prolific.com). A quota sampling strategy was used to attain sufficient variability in the primary dependent variable (i.e., persecutory beliefs). A pool of individuals representative of the UK population regarding age and gender was invited for the screening. Nonetheless, due to the quota sampling, the final sample is not representative of the general population. |
| Sampling strategy | The study used a quota sampling strategy by screening participant before inviting them to the main survey. A gender-balanced pool of participants who provided informed consent, were at least 18 years old, fluent in English, and currently living in the United Kingdom was invited to participate in the online screening. Only participants with a history of providing good-quality responses (i.e., an acceptance rate of ≥ 95%) were invited to ensure high data quality. Eligible participants were able to access the screening through their Prolific dashboard. Based on the screening, participants were assigned to one of four quotas according to their persecutory beliefs score (Revised Green Paranoid Thoughts Scale Persecution): average (< 5), elevated (6 - 10), moderate (11 - 17), and severe or very severe (≥ 18). If eligible, participants received invitations to the main survey within 24 hours after they had participated in the screening.<br>Since there are, to our knowledge, no validated approaches for sample size calculations for machine learning models, we based our estimation on a power analysis for multiple regression with medium effect size (f2 = .15) and high power (β = .95) using G*Power (version 3.1.9.7; Faul et al., 2009). This resulted in a final sample of N = 336 (n = 84 per quota). |
| Data collection | The data was collected in an online survey using the open-source platform PsyToolkit. |
| Timing | Data collection started on 1st of September 2022 and was completed on 16th of September 2022 |
| Data exclusions | In the Beads Estimation Task, data from participants with an error rate on the final decision exceeding 32% were excluded to ensure good data quality. Data from participants failing more than two of the four attention checks throughout the main survey were excluded (n = 1). Further data from n = 11 participants who provided incomplete data due to technical issues were excluded from the analyses. |
| Non-participation | Of n = 621 screened individuals who were invited to the main survey, n = 415 started the survey. Of these, n = 62 dropped out of the study before completion and n = 353 finished the survey. Eleven participants provided incomplete data and n = 1 participant failed the attention checks, resulting in the final sample of N = 336 participants. |

| | |
|---|---|
| Randomization | There were no experimental groups involved in this study. |

# Reporting for specific materials, systems and methods

We require information from authors about some types of materials, experimental systems and methods used in many studies. Here, indicate whether each material, system or method listed is relevant to your study. If you are not sure if a list item applies to your research, read the appropriate section before selecting a response.

## Materials & experimental systems

| n/a | Involved in the study |
|---|---|
| x | Antibodies |
| x | Eukaryotic cell lines |
| x | Palaeontology and archaeology |
| x | Animals and other organisms |
| x | Clinical data |
| x | Dual use research of concern |
| x | Plants |

## Methods

| n/a | Involved in the study |
|---|---|
| x | ChIP-seq |
| x | Flow cytometry |
| x | MRI-based neuroimaging |

## Plants

| | |
|---|---|
| Seed stocks | *Report on the source of all seed stocks or other plant material used. If applicable, state the seed stock centre and catalogue number. If plant specimens were collected from the field, describe the collection location, date and sampling procedures.* |
| Novel plant genotypes | *Describe the methods by which all novel plant genotypes were produced. This includes those generated by transgenic approaches, gene editing, chemical/radiation-based mutagenesis and hybridization. For transgenic lines, describe the transformation method, the number of independent lines analyzed and the generation upon which experiments were performed. For gene-edited lines, describe the editor used, the endogenous sequence targeted for editing, the targeting guide RNA sequence (if applicable) and how the editor was applied.* |
| Authentication | *Describe any authentication procedures for each seed stock used or novel genotype generated. Describe any experiments used to assess the effect of a mutation and, where applicable, how potential secondary effects (e.g. second site T-DNA insertions, mosiacism, off-target gene editing) were examined.* |
